# Supplementary figures and images for: A conserved trypanosomatid differentiation regulator controls substrate attachment and morphological development in Trypanosoma congolense
Source: PLoS Pathog. 2024 Feb 26;20(2):e1011889. doi: 10.1371/journal.ppat.1011889 (PMC10919850; doi:10.1371/journal.ppat.1011889)

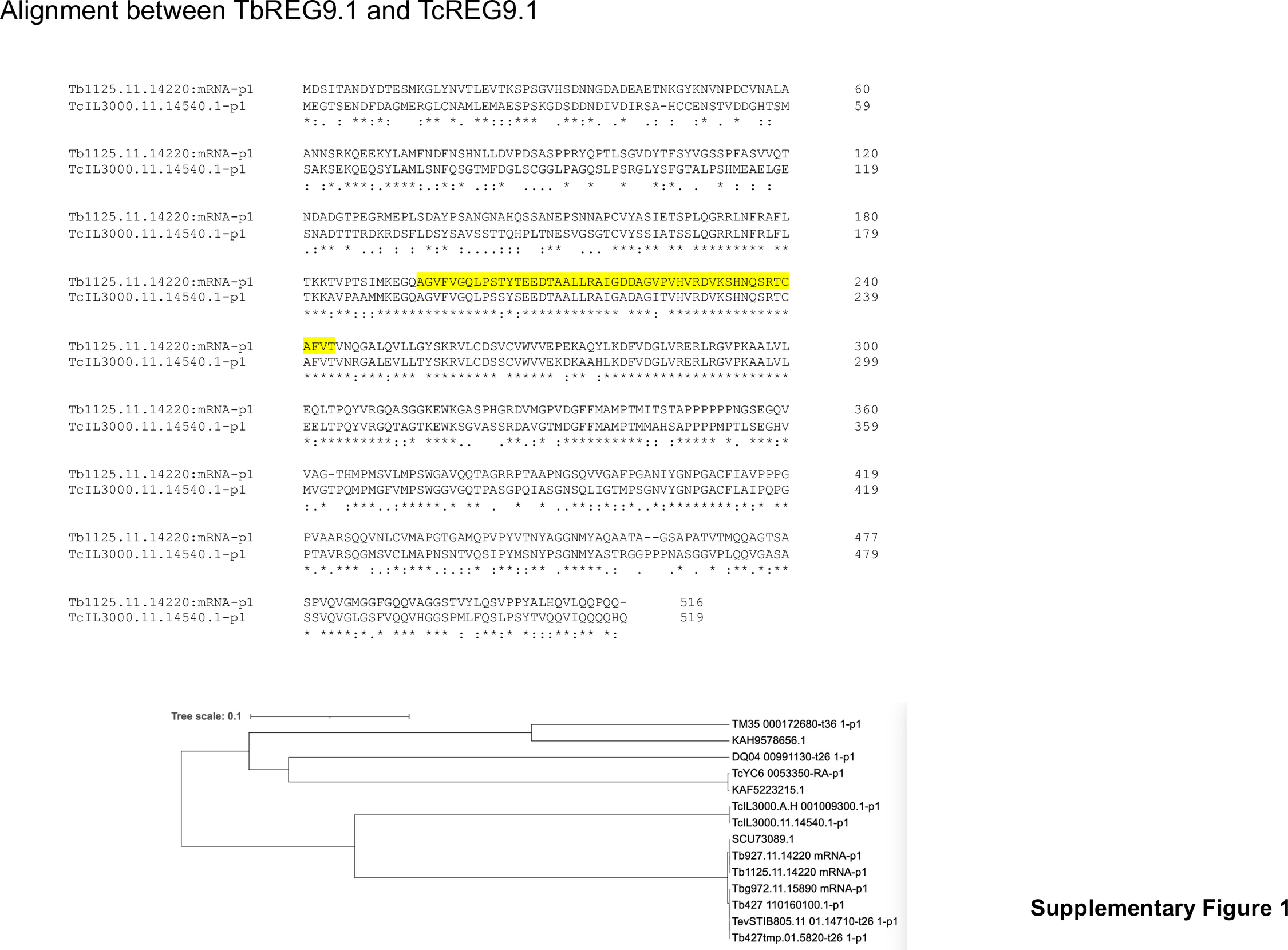

Supplement: S1 Fig — The TcREG9.1 gene encodes a 519 amino acid protein with an RRM predicted RNA binding domain positioned centrally (yellow shading). The phylogenetic relationship between REG9.1 orthologues in different kinetoplastids is shown below the alignment. (TIF) [file ppat.1011889.s001.tif]

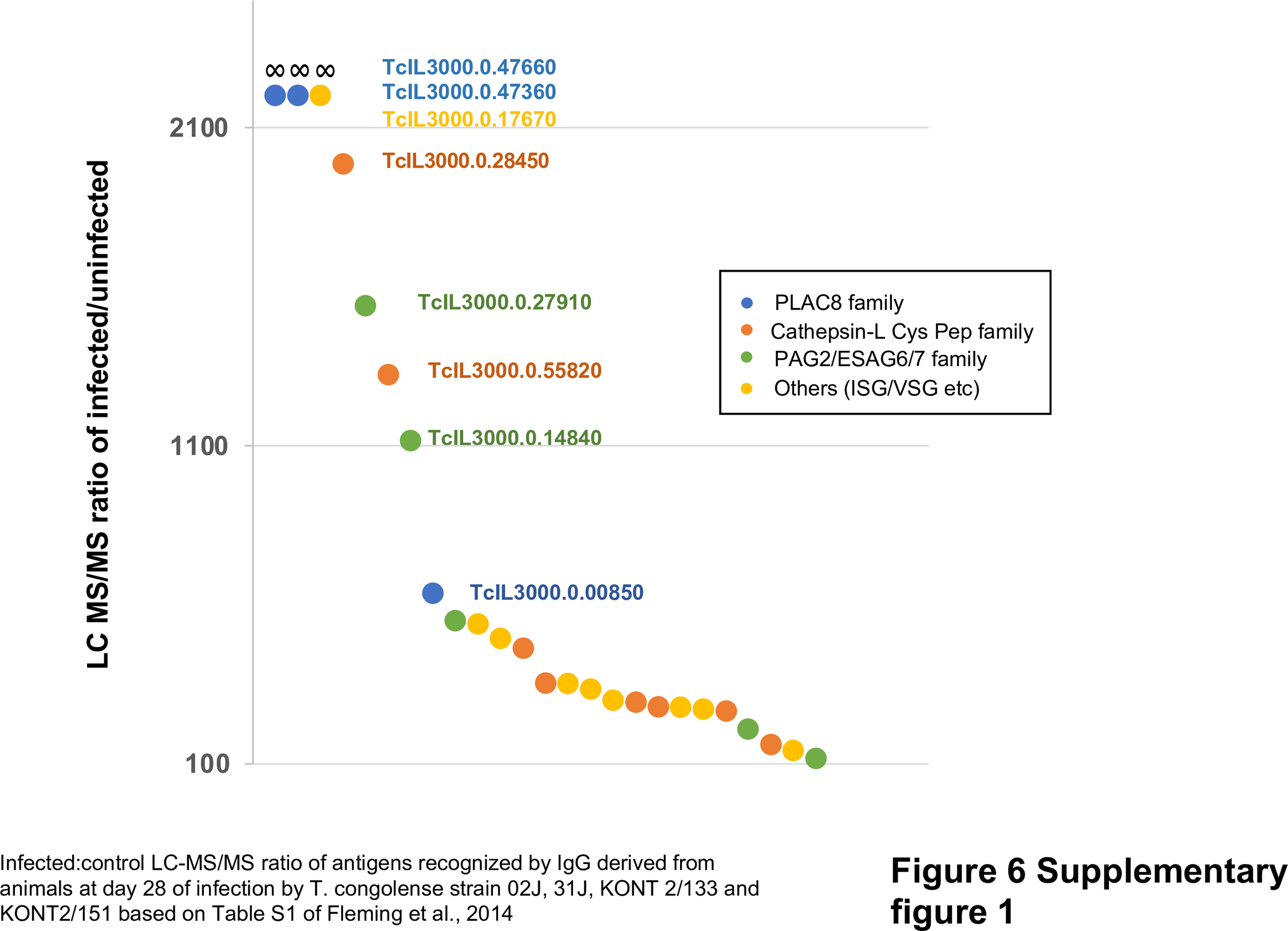

Supplement: S2 Fig — Individual transcripts are colour coded according to their encoded protein family, with the gene code for the most differentially detected annotated. (TIF) [file ppat.1011889.s002.tif]

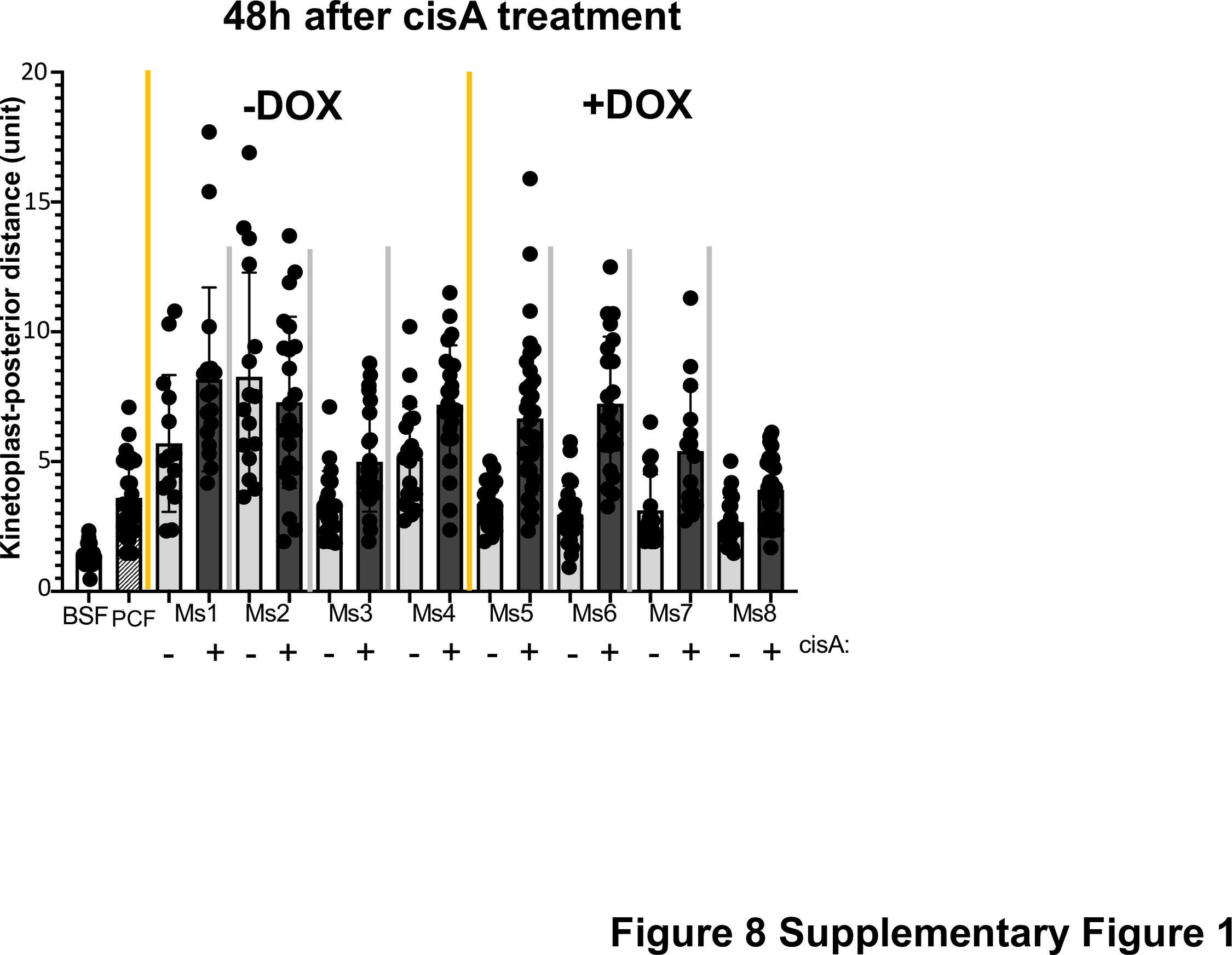

Supplement: S3 Fig — For each infection, harvested parasites were exposed or not to 6mM cis aconitate and the kinetoplast to posterior dimension determined at 48hr. As controls, cultured bloodstream forms (BSF) and cultured procyclic forms (PCF) were also included. The depletion of TcREG9.1 by RNAi resulted in a reduced kinetoplast repositioning in each case in the absence of cis aconitate. (TIF) [file ppat.1011889.s003.tif]

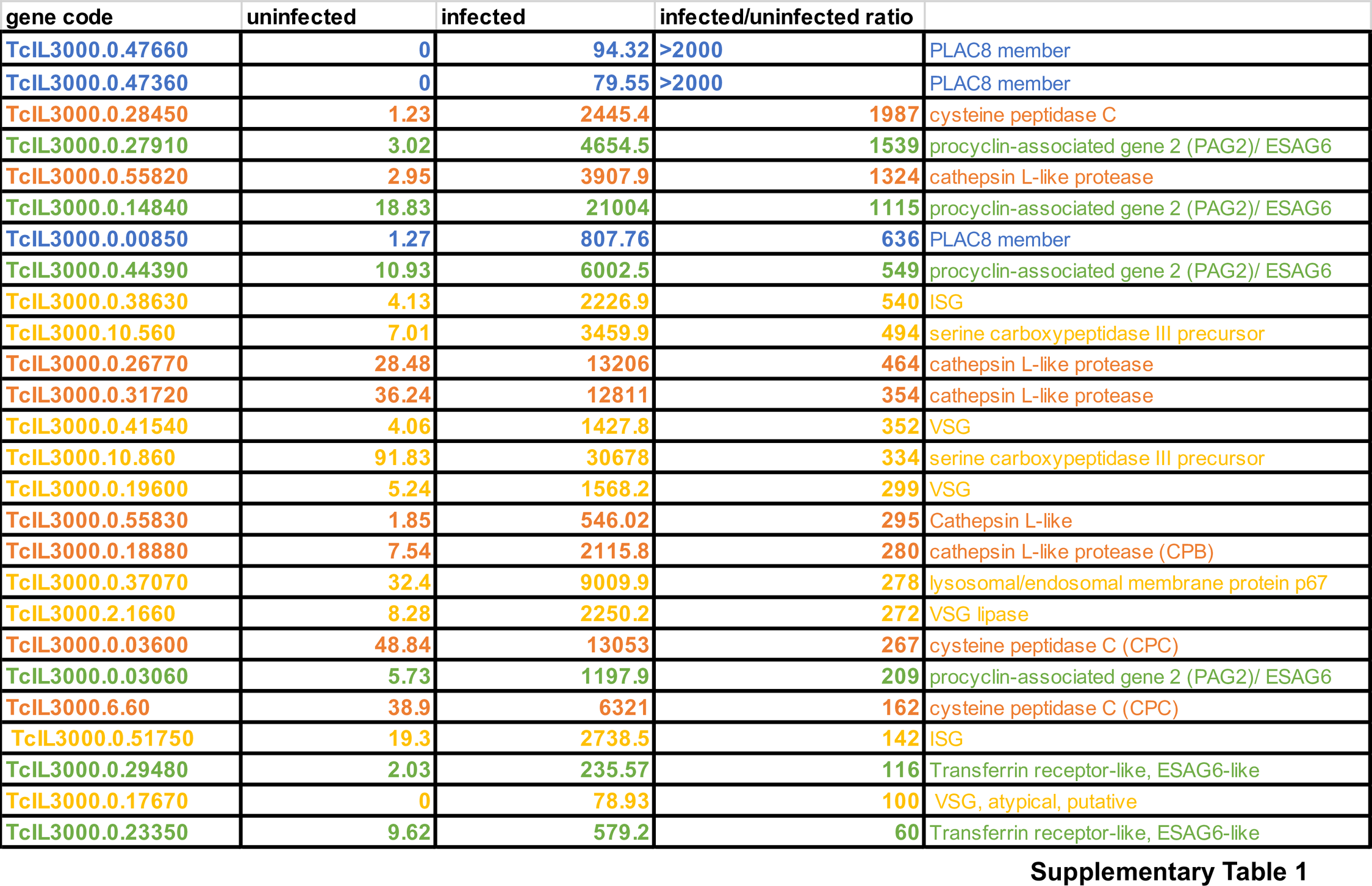

Supplement: S1 Table — Individual transcripts are colour coded according to their encoded protein family. (TIF) [file ppat.1011889.s004.tif]
