# Supplementary material for: A conserved trypanosomatid differentiation regulator controls substrate attachment and morphological development in Trypanosoma congolense
Source: PLoS Pathog. 2024 Feb 26;20(2):e1011889. doi: 10.1371/journal.ppat.1011889 (PMC10919850; doi:10.1371/journal.ppat.1011889)
Supplement: Attachment — Submitted filename: Silvester, Szoor et al, response to referees.docx [file ppat.1011889.s007.docx]

The manuscript was considered important and interesting by all reviewers and it requires only minor revisions/clarifications. Please take into consideration these comments and resubmit a new version.

We were very pleased each of the referees found the work important and interesting. We are happy to clarify their distinct comments on a point-by-point basis below

Reviewer Comments (if any, and for reference):

Reviewer's Responses to Questions

**Part I - Summary**
Please use this section to discuss strengths/weaknesses of study, novelty/significance, general execution and scholarship.

Reviewer #1: This is an interesting and important study by Silvester and Szoor et al, demonstrating that TcREG9.1, an orthologue of the T. brucei TbREG9.1 which regulates ESAG9 expression and some aspects of differentiation in T. brucei, plays a similar role in T. congolense, despite the absence of a morphologically distinct transmission-adapted stage in this parasite species. Moreover, they show that detachment from the microvasculature plays an important role in facilitating transmission for this parasite. Overall, this work represents an important advance in understanding how T. congolense adapts for transmission and the conserved nature of this process in African trypanosomes. The experiments are straightforward and well-controlled. I only have minor comments, primarily relating to the clarity of the discussion around the RNA-seq data. These are outlined below.

Reviewer #2: SYNOPSIS: Authors identify a T congolense homologue of REG9.1, a regulator of development in T brucei. They find that KD (~50% mRNA levels) of TcREG9.1 reduced growth rate, caused increased motility and reduced attachment to substrate surface in vitro. Noteably, the attachment and motility changes were phenocopied by PDE inhibition, indicating involvement of cAMP, as reported for attachment of the related parasite, Crithidia. RNAseq analysis revealed that TcREG9.1 depletion leads to misregulation of genes associated with T congolense development and parasite density-dependent responses in the mammalian bloodstream. Analysis of mouse-derived parasites corroborated this finding by revealing that some changes in cell morphological markers are TcREG9.1-dependent. Finally, the number of circulating parasites in an animal infection model was also increased in the TcREG9.1 KD, interpreted as consistent with anticipated reduced attachment to blood vessels.

CRITIQUE: This work addresses an important pathogen, T. congelense, that is much less studied than the related T. brucei and therefore provides considerable novelty and significance. The work is done rigorously, with suitable replicates, including independent isolates of the KD line, and the conclusions are well-supported. The paper is written clearly and concisely. I only have a few minor questions/comments for the authors to consider:

QUESTIONS/COMMENTS:
• Growth curves indicate growth subsides, then returns to approximately control growth rate. Does this reflect RNA returning to control levels, or parasite adapting to reduction of mRNA in the KD?

Thank you for this comment. In fact, we have only focused on the onset of the phenotype being characterised. Our experience with other gene knockdowns causing growth effects is that secondary adaptations (including resistance to RNAi) are common and that it is important to focus on the onset of phenotype to avoid indirect effects complicating analyses. Hence, the Referee has offered the most likely explanation but we have not explored it.

• Fig 2. I didn't see description of how % parasites in supernatant was determined. I imagine one can count parasites in the supernatant, but what method is used to count attached parasites to get the total number?

This is a good point and so we have clarified our scoring methodology in the Methods section (line 673-679)

• Regarding impact of PDE-inhibition on attachment - can the authors distinguish an effect on attachment, per se, versus an indirect effect, namely reduced attachment as a result of more vigorous motility? If not, text should be adjusted to indicate this. Note also that there is work in T brucei indicating loss of PDE results in reduced turning and greater straight-line velocity [Shaw et al. 2019 Nat Commun 10, p803], perhaps analogous to the increased motility seen for T. congolense here.

In fact, we discussed exactly this point in the original manuscript submission and also referenced the earlier work on T. brucei motility (albeit a review rather than the source manuscript). Perhaps the Referee missed it? It is on lines 384-392 of the revised annotated manuscript.

• For RNAseq analysis:
○ -- were these analyses done on detached cells only, or the entire population?

This was done on the whole population- accessible after harvesting the suspended cells and flushing the attached cells from the flask- as described earlier. We have now clarified this point in the methods (line 692)

○ -- I see logFC threshold. It would be helpful to include the adj p-value threshold (per leged) on the figure itself.

As the legend for the RNAseq dataset scatterplot in Figure 5 showed in the original manuscript, the datapoints presented were only those achieving the relevant adjusted P value (i.e. p<0.05). On Figure 6 we show (in different colours) all surface phylome family members detected, with those achieving an adjusted P value (p<0.05) threshold coloured differently to those that did not. We think this is a fair way to represent the full data set and the statistical relevance for each datapoint.

• Regarding anticipated surface location based on surface phylome annotation, the authors might also consider cross-referencing with the stage-dependent surface proteome analysis done in T. brucei [Shimogawa et al. (2015)Mol Cell Proteomics. 14, p1977], which looks at surface proteins directly, rather than predicting surface location via sequence analysis.

We have not done this because many of the relevant regulated molecules are T. congolense specific- as detailed in the original surface phylome classifications.

• Fig 7: The figure legend is unclear.
○ Does each plot correspond to a different animal? Yes. We have now been explicit in the legend.

○ It would add clarity to indicate "Uninduced" and "Induced" directly on panel a and b, respectively. Done

○ Update the 7b legend to change ndicates a and b are (a) vs "TcREG9.1 cell line" to "TcREG9.1 RNAi cell line".

We are not quite sure what the Referee is requesting, but we believe the legend was clear and so have not changed it.

• Regarding Fig 8.
○ Panel C: I would recommend kp-posterior dimension cartoon trypanosomes be black, since the data in that chart do not reflect PRS expression, which I interpret to be represented by red vs blue outline. Likewise, the PRS panel should exclude the nucleus and kp position, as those are not reflected in the data shown there. It would also be helpful to explain this in the figure legend, which currently does not mention the cartoon trypanosomes.

We apologise- the cartoons were included to clarify the message from the Figure data, with the intention to help highlight the salient observation. They were explained in the panel a legend, but we did not repeat the same information for each panel. Clearly this wasn’t successful! Therefore, we have taken the Referee’s suggestion and used black and white presentations, and removed these from the PRS panels. We have also reordered the data columns to make the relevant comparisons clearer (as detailed in below).

○ It would be useful (though not required) if the authors did this experiment looking at parasite attachment directly, as done in the orginal Banks paper. I agree, however, that the experiment as done would appear to provide an indirect proxy for estimating attachment. Looking back at the abstract, they phrase the interpretation appropriately.

Given the scale and difficult of the experiment we have opted not to repeat it with an alternative scoring method. As the Referee points out, we have been careful to accurately describe our interpretation in the text (and Abstract).

§ NOTE: Has a similar analysis been done with T. brucei? If the difference between TS and CP reflects attachment to vessels exclusively, the ratio should approximate unity for T. brucei, correct?

We have not included a similar analysis with T. brucei - our focus was on T. congolense. However, we can confirm from many independent experiments in our laboratory that T. brucei parasite recovery from cardiac blood accurately maps to the parasitaemia determined by tail snip, consistent with the reported absence of adherence in T. brucei.

○ For the claim on p13 that "…mouse derived parasites responded to cis aconitate after 24h by increasing their kinetoplast posterior dimension", please provide statistical analysis of the 24hr data on kp-posterior distance.

P values have now been included in the text and figure. Also a supplementary Table has been included detailing the statistical values of all pairwise comparisons in the Figure (Figure 8 supplementary Table 1).

Reviewer #3: The experiments in the manuscript build on those using Trypanosoma brucei that have shown that there is a clear developmental transition in bloodstream form trypanosomes from proliferative to a morphologically distinct cell cycle arrested stumpy form. These experiments identified mRNAs that are increased in stumpy forms including REG9.1 which encodes a protein containing a RRM motif, often but not always involved in RNA binding.

Here, the T. congolense homologue of REG9.1 is analysed and RNAi-mediated depletion shown to alter the ability to sequester onto surfaces. Cell lines were constructed to knock down REG9.1 mRNA and resulted in a ~50% decrease. The most noticeable phenotype was a reduction in adherence. This was phenocopied by a phosphodiesterase inhibitor which also altered the motility (distance travelled). The experiments go on to characterise changes in mRNA levels after 3 days of RNAi and provide strong evidence it represents a similar pathway to that present in T. brucei.

Overall, the experiments show that a similar developmental pathway regulates stumpy formation in T. brucei and adherence in T. congolense. It is a good piece of work.

Thank you for the positive comments

**Part II – Major Issues: Key Experiments Required for Acceptance**
Please use this section to detail the key new experiments or modifications of existing experiments that should be absolutely required to validate study conclusions.

Generally, there should be no more than 3 such required experiments or major modifications for a "Major Revision" recommendation. If more than 3 experiments are necessary to validate the study conclusions, then you are encouraged to recommend "Reject".

Reviewer #1: (No Response)

Reviewer #2: NA

Reviewer #3: none

**Part III – Minor Issues: Editorial and Data Presentation Modifications**
Please use this section for editorial suggestions as well as relatively minor modifications of existing data that would enhance clarity.

Reviewer #1: Figure 6 - The discussion around Figure 6 was hard to follow for me. Family 22 is highlighted but expression is really variable; how is this supposed to be interpreted?

There are many representatives of surface phylome family 22 with variable expression in response to TcREG9.1 RNAi. However, focusing only on those with a significant adjusted P value (green on the upper panel of Figure 6 from clone 3.3, blue for the lower panel from clone 4.5) most members are upregulated and these are among the most upregulated surface phylome family of any analysed. Given that Family 22 members were also identified as upregulated in peak parasitaemia T. congolense mouse infections (Silvester et al., 2018; PLOS NTD, PMID: 30307943), this reinforces the role of TcREG9.1 in regulating developmental transcripts.

I was also confused by the focus on immunodominant antigens?. There could be more discussion of what this might mean. Why would transmission associated antigens be immunodominant?

We describe these families as immunodominant because antisera from cows infected with T. congolense immunoslects members of these protein families at the highest levels as detected by mass spectrometry. This data was derived from the published study from Mike Ferguson’s group (Fleming et al 2014; PMID: 24922510), the relative enrichment being shown in Figure 6 supplementary Figure 1. We interpret this observation as indicating the molecules are expressed as proteins by the parasite (allowing antisera to recognise them) and the level of recognition indicated they are either highly expressed (and so clearly recognised) or highly immunogenic. Being the most enriched families among the proteins detected by cows led us to describe them as immunodominant although ‘immunoreactive’ may be more accurate - and so we have amended our text to use this phraseology. The detection of these proteins – elevated in parasites that have developed toward a potential developmental form adapted for transmission- could be because detached parasites are more likely to be immunogenic, or less effective at immune evasion. These are questions we are keen explore in future.

Moreover, the variability among members of families that are highlighted is confusing. As it is, I am not sure what this analysis adds to the manuscript. Therefore, I'd recommend more discussion/context to make the implications of the highlighted findings absolutely clear. Looking at the discussion, it appears the major observation is that surface proteins generally are upregulated after knockdown. Perhaps some statistical analysis would show they are disproportionately represented in the upregulated gene set?

As highlighted above the Figure shows that the expression of certain surface phylome families are enriched with TcREG9.1 depletion. Our discussion has focused on those with highest fold enrichment and statistical significance. We have tried to improve our text to improve clarity.

Figure 8 - While everything is in this figure, I found it hard to link the data in the figure to the descriptions in the text. There may be ways to reorganize the visualization so that the pertinent comparisons are highlighted and more obvious to a reader (for example, putting samples/conditions you explicitly contrast next to one another on the graph). This is not a major issue, but it did take me a while to parse this particular figure.

We tried to highlight the most important effect of the TcREG9.1 depletion (i.e. an effect on kinetoplast positioning in the absence of cis aconitate) with the annotated purple arrows. Nonetheless we have now reconfigured the plots to make the relevant comparisons easier to identify by re-ordering the columns. We have also restricted the use of schematic representations of the kinetoplast repositioning to only those panels where it is relevant and clear (t=0; t=48h) and removed the colour representation of PRS expression as suggested. We have not changed the column ordering etc on Figure 8 supplementary 1 because here it is appropriate to keep the data (± cis aconitate) from the individual mice adjacent.

Figure 9 - I assume KP stands for kinteoplast position. The authors could spell this out on the figure or in the legend. Done

Lines 84-85 - It may be more clear to state that the two morphological forms mentioned are in the mammalian host. This is mentioned in later sentences but I think better to state up front. Done

Line 113 - RRM is defined eventually on line 389 but should probably be defined at the first mention Done

Line 174 - typo, I think you mean "knock down" Thank you for spotting this!

Reviewer #2: See Summary, "Questions/Comments"

Reviewer #3: Define ‘immunodominant’ in abstract. Experimental rather than annotation and animal in which they are immunodominant. Amended

Line 180
Comment on whether the reduction in growth is transient for ~3 days Done

Line 212
Comment on high concentration of PDE inhibitor, was it the same as used for the experiments in Crithidia? Yes it was the same as used in Crithidia (although of course we tried several concentrations).

PLOS authors have the option to publish the peer review history of their article ([what does this mean?](https://eur02.safelinks.protection.outlook.com/?url=https%3A%2F%2Fjournals.plos.org%2Fplospathogens%2Fs%2Feditorial-and-peer-review-process%23loc-peer-review-history&data=05%7C02%7Ckeith.matthews%40ed.ac.uk%7C2b5fe3c6a03d4e2a8da308dc21193a71%7C2e9f06b016694589878910a06934dc61%7C1%7C0%7C638421639438046463%7CUnknown%7CTWFpbGZsb3d8eyJWIjoiMC4wLjAwMDAiLCJQIjoiV2luMzIiLCJBTiI6Ik1haWwiLCJXVCI6Mn0%3D%7C0%7C%7C%7C&sdata=LTTLHD%2FmSZeQ1KZCQbhgkJoJZ8%2B081S2Doz6NT%2FntnE%3D&reserved=0)). If published, this will include your full peer review and any attached files.


If you choose “no”, your identity will remain anonymous but your review may still be made public.


**Do you want your identity to be public for this peer review?** For information about this choice, including consent withdrawal, please see our [Privacy Policy](https://eur02.safelinks.protection.outlook.com/?url=https%3A%2F%2Fwww.plos.org%2Fprivacy-policy&data=05%7C02%7Ckeith.matthews%40ed.ac.uk%7C2b5fe3c6a03d4e2a8da308dc21193a71%7C2e9f06b016694589878910a06934dc61%7C1%7C0%7C638421639438063035%7CUnknown%7CTWFpbGZsb3d8eyJWIjoiMC4wLjAwMDAiLCJQIjoiV2luMzIiLCJBTiI6Ik1haWwiLCJXVCI6Mn0%3D%7C0%7C%7C%7C&sdata=T3cLQW%2B3RpuO8zL4ggb5HhQdaPPeLjpOt8jmqu1SyJc%3D&reserved=0).

Reviewer #1: No

Reviewer #2: **Yes:** Kent L. Hill

Reviewer #3: No

Figure Files:

While revising your submission, please upload your figure files to the Preflight Analysis and Conversion Engine (PACE) digital diagnostic tool, <https://pacev2.apexcovantage.com>. PACE helps ensure that figures meet PLOS requirements. To use PACE, you must first register as a user. Then, login and navigate to the UPLOAD tab, where you will find detailed instructions on how to use the tool. If you encounter any issues or have any questions when using PACE, please email us at [figures@plos.org](mailto:figures@plos.org).

Data Requirements:

Please note that, as a condition of publication, PLOS' data policy requires that you make available all data used to draw the conclusions outlined in your manuscript. Data must be deposited in an appropriate repository, included within the body of the manuscript, or uploaded as supporting information. This includes all numerical values that were used to generate graphs, histograms etc.. For an example see here: [http://www.plosbiology.org/article/info%3Adoi%2F10.1371%2Fjournal.pbio.1001908#s5](https://eur02.safelinks.protection.outlook.com/?url=http%3A%2F%2Fwww.plosbiology.org%2Farticle%2Finfo%253Adoi%252F10.1371%252Fjournal.pbio.1001908%23s5&data=05%7C02%7Ckeith.matthews%40ed.ac.uk%7C2b5fe3c6a03d4e2a8da308dc21193a71%7C2e9f06b016694589878910a06934dc61%7C1%7C0%7C638421639438071558%7CUnknown%7CTWFpbGZsb3d8eyJWIjoiMC4wLjAwMDAiLCJQIjoiV2luMzIiLCJBTiI6Ik1haWwiLCJXVCI6Mn0%3D%7C0%7C%7C%7C&sdata=bQ9YiVn%2BFfoleDAh57kxnYoEVUWzjsAXCOi39Q0oKzM%3D&reserved=0).

Reproducibility:

To enhance the reproducibility of your results, we recommend that you deposit your laboratory protocols in [protocols.io](http://protocols.io), where a protocol can be assigned its own identifier (DOI) such that it can be cited independently in the future. Additionally, PLOS ONE offers an option to publish peer-reviewed clinical study protocols. Read more information on sharing protocols at[https://plos.org/protocols?utm_medium=editorial-email&utm_source=authorletters&utm_campaign=protocols](https://eur02.safelinks.protection.outlook.com/?url=https%3A%2F%2Fplos.org%2Fprotocols%3Futm_medium%3Deditorial-email%26utm_source%3Dauthorletters%26utm_campaign%3Dprotocols&data=05%7C02%7Ckeith.matthews%40ed.ac.uk%7C2b5fe3c6a03d4e2a8da308dc21193a71%7C2e9f06b016694589878910a06934dc61%7C1%7C0%7C638421639438077888%7CUnknown%7CTWFpbGZsb3d8eyJWIjoiMC4wLjAwMDAiLCJQIjoiV2luMzIiLCJBTiI6Ik1haWwiLCJXVCI6Mn0%3D%7C0%7C%7C%7C&sdata=%2BCj%2FHNdTEKz75AlbQYZ4uRSmWFYCiBeO2SJ%2BAmII12E%3D&reserved=0)

References:

Please review your reference list to ensure that it is complete and correct. If you have cited papers that have been retracted, please include the rationale for doing so in the manuscript text, or remove these references and replace them with relevant current references. Any changes to the reference list should be mentioned in the rebuttal letter that accompanies your revised manuscript. If you need to cite a retracted article, indicate the article’s retracted status in the References list and also include a citation and full reference for the retraction notice.

*In compliance with data protection regulations, you may request that we remove your personal registration details at any time.* [*(Remove my information/details)*](https://eur02.safelinks.protection.outlook.com/?url=https%3A%2F%2Fwww.editorialmanager.com%2Fppathogens%2Flogin.asp%3Fa%3Dr&data=05%7C02%7Ckeith.matthews%40ed.ac.uk%7C2b5fe3c6a03d4e2a8da308dc21193a71%7C2e9f06b016694589878910a06934dc61%7C1%7C0%7C638421639438083922%7CUnknown%7CTWFpbGZsb3d8eyJWIjoiMC4wLjAwMDAiLCJQIjoiV2luMzIiLCJBTiI6Ik1haWwiLCJXVCI6Mn0%3D%7C0%7C%7C%7C&sdata=YQ%2FZQ5qCd3VFEplyU%2BwVFVnSGWN2YNeVfZysg8cjy2o%3D&reserved=0)*. Please contact the publication office if you have any questions.*
